# An *In silico *approach for the evaluation of DNA barcodes

**DOI:** 10.1186/1471-2164-11-434

**Published:** 2010-07-16

**Authors:** Gentile Francesco Ficetola, Eric Coissac, Stéphanie Zundel, Tiayyba Riaz, Wasim Shehzad, Julien Bessière, Pierre Taberlet, François Pompanon

**Affiliations:** 1 Laboratoire d'Ecologie Alpine, CNRS UMR 5553, Université Joseph Fourier, BP 53, F-38041 Grenoble Cedex 9, France; 2 Dipartimento di Biologia, Università degli Studi di Milano. Via Celoria 26, 20133 Milano Italy; 3 Dipartimento di Scienze dell'Ambiente e del Territorio, Università degli Studi di Milano Bicocca. Piazza della Scienza 1, 20126 Milano Italy

## Abstract

**Background:**

DNA barcoding is a key tool for assessing biodiversity in both taxonomic and environmental studies. Essential features of barcodes include their applicability to a wide spectrum of taxa and their ability to identify even closely related species. Several DNA regions have been proposed as barcodes and the region selected strongly influences the output of a study. However, formal comparisons between barcodes remained limited until now. Here we present a standard method for evaluating barcode quality, based on the use of a new bioinformatic tool that performs *in silico *PCR over large databases. We illustrate this approach by comparing the taxonomic coverage and the resolution of several DNA regions already proposed for the barcoding of vertebrates. To assess the relationship between *in silico *and *in vitro *PCR, we also developed specific primers amplifying different species of Felidae, and we tested them using both kinds of PCR

**Results:**

Tests on specific primers confirmed the correspondence between *in silico *and *in vitro *PCR. Nevertheless, results of *in silico *and *in vitro *PCRs can be somehow different, also because tuning PCR conditions can increase the performance of primers with limited taxonomic coverage. The *in silico *evaluation of DNA barcodes showed a strong variation of taxonomic coverage (i.e., universality): barcodes based on highly degenerated primers and those corresponding to the conserved region of the *Cyt*-b showed the highest coverage. As expected, longer barcodes had a better resolution than shorter ones, which are however more convenient for ecological studies analysing environmental samples.

**Conclusions:**

*In silico *PCR could be used to improve the performance of a study, by allowing the preliminary comparison of several DNA regions in order to identify the most appropriate barcode depending on the study aims.

## Background

DNA barcoding, i.e., the identification of biological diversity using standardized DNA regions, has been demonstrated as a new, very useful approach to identify species [1]. Originally, DNA barcoding was proposed to assign an unambiguous tag to each species, giving to taxonomists a standard method for identification of specimens. In this context, it was also proposed that DNA barcoding is an opportunity to accelerate the discovery of new species [2-4]. Today, the fields of applications of this approach are broader. As example, DNA barcoding has been already used in biodiversity assessment, forensics, diet analysis and paleoecological studies [5-7].

In the former context, a portion of mitochondrial cytochrome *c *oxidase (*COI*) has been proposed as the standard barcode for animal identification [1,8]. Since then, other portions of DNA have been proposed as barcodes, because different DNA regions have different performances in some taxa (e.g., flowering plants [9,10]; amphibians [11]). If we consider the other applications of barcoding (*sensu lato *DNA barcoding, [6]), the necessity to limit the number of usable barcode loci for conserving the standard aspect of this method can be relaxed. In such a new context, multiple barcodes in different regions of the genome could be combined to improve identification, according to the taxon studied and to the aims of the research [9,10]. Therefore, the first step of a *sensu lato *barcoding study should be the selection of the best DNA region(s) to be used as barcode considering the aims of the study. The availability of large public sequence databases may allow comparing multiple potential barcodes and their properties before performing studies.

Among the properties of an ideal DNA barcode, high taxonomic coverage and high resolution are essential [6,12]. A high taxonomic coverage (also called universality) would allow the application of barcodes to a number of taxa as large as possible, including undescribed species. This constraints the DNA barcode region to have sufficiently conserved flanking regions enabling the design of universal primers. This is especially important for describing unknown biodiversity or diversity within environmental samples such as soils or faeces [6,7,13]. However, universality can be extremely difficult to achieve, because of the incomplete knowledge of genetic variation in poorly studied taxa [12]. The resolution capacity of a barcode is its ability to differentiate and identify species that relies on interspecific differences among DNA sequences [8,14]. Thus, the challenge for defining a barcode of good quality consists in finding a quite short and enough variable DNA sequence flanked by highly conserved regions. Depending of the application, the size, the taxonomic coverage or the resolution of the DNA barcode could be the most important characteristic to optimise [6].

This study proposes an explicit approach for comparing the performance of potential barcoding regions, which is based on '*in silico *PCRs' performed over extensive databases, and on two indices that estimate the resolution capacity of the barcodes and the taxonomic coverage of the primers used for their amplification. As an example, we analysed several primers available from the literature that have been used in *sensu lato *barcoding studies [6] for the identification of Vertebrates species. First, we assessed the taxonomic coverage of several primer pairs by evaluating the proportion of species amplified *in silico *in a purposely designed database. Subsequently, we analyzed the GenBank sequences amplified by each primer pair, in order to evaluate the proportion of species correctly identified on the basis of their barcodes. We also used an *in vitro *analysis to validate the correspondence between *in silico *and real world PCR.

## Methods

### General strategy

First, we created a reference database representative of the mitochondrial genomes of all vertebrates, by retrieving from Genbank all the complete mitochondrial genomes of Vertebrates available (accession: September 2007). Subsequently, we randomly selected one sequence per species, to reduce the overrepresentation of a few species (e.g., humans, mouse, zebrafish etc.). We obtained a set of 814 mitochondrial genomes representative of the five major monophyletic clades of vertebrates [Chondrichthyes: 8 species; Actinopterigii: 385 species; Amphibia: 79 species; Sauropsida (= birds + "reptiles"): 133 species; Mammalia: 202 species; other taxa: 7 species]. Most of species were the unique representative of their genus and the database corresponded to 633 genera.

To analyze the performance of each primer pair studied, we first performed an *in silico *PCR on the reference database and we evaluated the taxonomic coverage of each primer pair as the proportion of amplified taxa. Then, we performed an *in silico *PCR on the whole GenBank, to evaluate the resolution of the amplified fragments that represents the proportion of unambiguously identified taxa. These properties were evaluated for the whole Vertebrates and for each of the five clades which compose it.

### *In Silico *PCR

An *in silico *PCR consists in selecting in a database the sequences that match (i.e., exhibit similarity with) two PCR primers. The regions matching the two primers should be localised on the selected sequence in a way allowing PCR amplification, which forces the relative orientation of the matches and the distance between them. In order to simulate real PCR conditions, the *in silico *PCR algorithm should allow some mismatches between the primers and the target sequences. Standard sequence similarity assessment programs such as BLAST [15] are not suitable for such kind of analysis because the heuristic search they use is not efficient on short sequences. Moreover, a post processing of BLAST output should be performed to verify previously stated constraints. We have developed a program named ecoPCR that is based on the very efficient pattern matching algorithm Agrep [16]. This algorithm allows specifying the maximum count of mismatched positions between each primer and the target sequence, and to use the full IUPAC code (e.g., R for purines or Y for pyrimidines). It also allows specifying on which primer's specific positions mismatches are not tolerated, what is useful to force exact match on the 3' end of primers for simulating real PCR conditions. Moreover, to facilitate further analysis, ecoPCR output contains the taxonomic information for each sequence selected from the database. For the analyses presented in this article, we allowed two mismatches between each primer and the template, except on the last 3 bases of the 3' end of the primer. Analyses performed with 0, 1 or 3 mismatches led to similar conclusions (results not shown), even if the results were sometimes different (see discussion). This software was developed for Unix platforms and is freely available at http://www.grenoble.prabi.fr/trac/ecoPCR.

### Measuring taxonomic coverage

To measure the taxonomic coverage of a primer pair, we defined a coverage index B_c _as the ratio between the number of amplified taxa for a specified taxonomic rank (i.e., species for this analysis; genus or family can be specified as alternative taxonomic ranks) and the total number of taxa of the same level representing the studied clade in the reference sequence database. B_c _can be computed from ecoPCR output file using the ecoTaxStat script.

### Measuring resolution capacity

**Figure 1:**
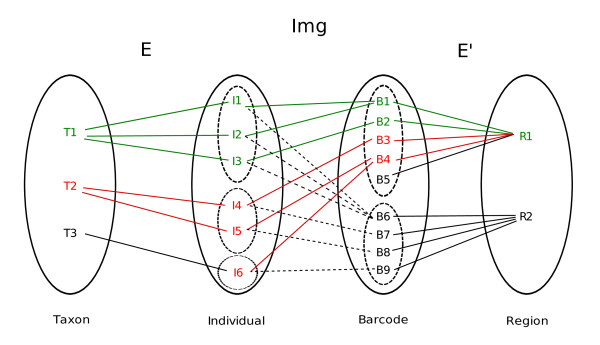
**Relationships between taxa, individuals, barcodes and regions as used in the B_s _index estimation**. In this example the taxon T1 is unambiguously identified by the R1 barcode region (green links) but the T2 is not well identified by the R1 region because this taxon share the B4 barcode region with the T3 taxon via the I6 individual (red links).

The resolution capacity of a barcode was estimated by an index measuring the ratio of unambiguously identified taxa for a given taxonomic level over the total number of tested taxa. A taxon unambiguously identified by a primer pair owns a barcode sequence associated to this pair that is not shared by any other taxa of the same taxonomic rank. To be computed, this definition can be formalized considering the mapping *E*, *Img *and *E' *between four concept sets: taxon (*T*), individual (*I*), barcode (*B*) and region (*R*) (for a full definition see figure 1). Considering the a taxon *t ∈ T *and a primer pair (barcode region) *r ∈ R *and using the mapping *E*, *Img *and *E' *we define the Ω(t,r) set of all barcodes belonging to a taxon for a region:

Ω(t,r) =Img(E(t)) ∩E'(r)

From the above description, we note the set of all individuals owning a barcode corresponding to a taxon as:

$$Img^{-1}(\Omega )\equiv \underset{i}{\cup }Img^{-1}(b_{i}/b_{i}\in \Omega )$$

This allows defining an unambiguously identified taxon t by a barcode region r if and only if:

$$Img^{-\text{1}}\left(\Omega (t,r))\;=E\left(t\right)\right)$$

This defines a mapping *ε *of *T *to *R *and allows to define the specificity index B_s _as:

$$B_{s}(r)=\frac{\left|\left\{t/t\varepsilon \;r\right\}\right|}{\left|T\right|}$$

B_s _can be computed from an ecoPCR output file using the ecoTaxSpecificity script. ecoTaxSpecificity and ecoTaxStat scripts are parts of the OBITools python package freely available at http://www.grenoble.prabi.fr/trac/OBITools.

In a few cases, especially for Chondrichthyes, ecoPCR ran over the entire GenBank yielded only a small number of sequences. Thus, we calculated the resolution capacity of a barcode only when the primer pair amplified more than 10 species.

### Correspondance between in vitro and in silico PCRs

Strict experimental validation of the electronic PCR realized over large databases would be extremely difficult, as it would require obtaining tissues from hundreds of species. Alternatively, specific primer pairs designed to amplify only one species can be used to confirm the correspondence between the results of ecoPCR and *in vitro *PCR. Therefore, we designed specific primers to amplify mitochondrial DNA of three species, using ecoPCR to test their specificity. Then, we cross-amplified the three species with each primer pairs with *in vitro *PCR to verify the ecoPCR predictions.

We considered three species of Asiatic Felidae: the Leopard (*Panthera pardus*); the Snow Leopard (*Uncia uncia*) and the Leopard cat (*Prionailurus bengalensis*). We designed specific primers for amplifying short sequences of mitochondrial 12S; this kind of primer pairs can be used to identify species from degraded DNA and remains, such as faeces. The three primer pairs were: (a) *Pant*F, 5'-GTCATACGATTAACCCGG-3'; *Pant*R, 5'-TGCCATATTTTTATATTAACTGC-3', designed to amplify the Leopard (amplified fragment: 120 bp); (b), *Unci*F, 5'-CTAAACCTAGATAGTTAGCT-3', *Unci*R, 5'-CTCCTCTAGAGGGGTG-3', designed to amplify the Snow Leopard (amplified fragment: 104 bp); (c) *Prio*F, 5'-CCTAAACTTAGATAGTTAATTTT-3', *Prio*R, 5'-GGATGTAAAGCACCGCC-3', designed to amplify the Cat Leopard (amplified fragment: 94 bp). DNA was extracted from faeces using QiAamp DNA Stool Kit (Qiagen GmbH, Hilden, Germany). The PCRs were conducted in a 20 μl total volume with 8 mM Tris-HCl (pH 8.3), 40 mM KCl, 2 mM MgCl2, 0.2 μM of each primer, BSA (5 μg), 0.5 U of AmpliTaq Gold DNA polymerase (Applied Biosystems) and 2 ml of DNA extract. For all primers, the PCR programme included an initial 10 min denaturation step at 95°C, 45 cycles of denaturation at 95°C for 30 s and annealing at 53°C for 30 s. Samples of each of the three species were amplified with the three primer pairs, to verify *in vitro *the possibility of cross-amplification. We also tested cross-amplification ability of these primer pairs using ecoPCR, allowing two mismatches between each primer and the template, except on the last 3 bases of the 3' end of the primer; subsequently, we simulated more relaxed PCR conditions [17] by allowing a larger number of mismatches.

### Vertebrate primer pairs tested

The vertebrate primers tested (table 1) were selected in the bibliography as representative of the diversity of the strategies used for defining barcodes. Some of them (COI-1, COI-2, COI-3) were highly degenerated, in order to maximise the number of taxa amplified (i.e., the taxonomic coverage) [18]. Most of primers chosen amplified long sequences (> 500 bp) to maximize resolution, while some (e.g., Uni-Minibar, 16Smam) have been designed to amplify short sequences, to maximize the possibility of retrieving sequences from damaged/ancient DNA [19-21].

**Table 1:** Vertebrate primer pairs tested.

Barcode name	Primer Name	Sequence	Fragment size *	Developed for	Reference
**COI**					
COI-1	FF2d	TTCTCCACCAACCACAARGAYATYGG	655	Fish	[18]
	FR1d	CACCTCAGGGTGTCCGAARAAYCARAA			
COI-2H	LCO1490	GGTCAACAAATCATAAAGATATTGG	658	mainly Arthropods	[1]
	HCO2198	TAAACTTCAGGGTGACCAAAAAATCA			
COI-2	C_VF1LFt1	WYTCAACCAAYCANAANGANATNGG	658	Fish	[18]; modified from [1]
	C_VR1LRt1	TARACTTCTGGRTGNCCNAANAANCA			
COI-3	C_FishF1t1	TCRACYAAYCAYAAAGAYATYGGCAC	652	Fish	[18]
	C_FishR1t1	ACYTCAGGGTGWCCGAARAAYCARAA			
Uni-Minibar	UniMinibarR1	GAAAATCATAATGAAGGCATGAGC	130	Eukaryota	[20]
	UniMinibarF1	TCCACTAATCACAARGATATTGGTAC			

**Cyt-*b***					
MCB	mcb398	TACCATGAGGACAAATATCATTCTG	472	All Vertebrates	[30]
	mcb869	CCTCCTAGTTTGTTAGGGATTGATCG			
cytM	L14841	CCATCCAACATCTCAGCATGATGAAA	359	All Vertebrates	[31]; modif. from [26]
	H15149	CCCCTCAGAATGATATTTGTCCTCA			

**16S**					
16Sr	16Sar	CGCCTGTTTATCAAAAACAT	573	Mammals	[27,28]
	16Sbr	CCGGTCTGAACTCAGATCACGT			
16Sr2	16Sa2	CGCCTGTTTACCAAAAACAT	573	All Vertebrates	this study, modif. from [28]
	16Sb	CCGGTCTGAACTCAGATCACGT			
16Smam	16Smam1	CGGTTGGGGTGACCTCGGA	140	Mammals, ancient DNA	[21]
	16Smam2	GCTGTTATCCCTAGGGTAACT			

* as reported on the original paper.

## Results

### Validation of in silico PCR

**Figure 2:**
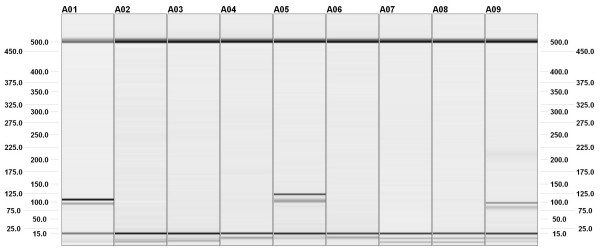
**Capillary electrophoresis (QIAxcel System, Qiagen) showing the results of cross amplification of three species of Felidae using three specific primers**. A01: *Unci *primers, template DNA from *Uncia uncia*; A02: *Unci *primers, template DNA from *Panthera pardus*; A03: *Unci *primers, template DNA from *Prionailurus bengalensis*; A04: *Pant *primers, template DNA from *U. uncia*; A05: *Pant *primers, template DNA from *P. pardus*; A06: *Pant *primers, template DNA from *P. bengalensis*; A07: *Prio *primers, template DNA from *U. uncia*; A08: *Prio *primers, template DNA from *P. pardus*; A09: *Prio *primers, template DNA from P. bengalensis. The size in base pairs is indicated on the left and on the right.

With *in vitro *PCR, each pair of specific primers amplified only the species for which it was designed: *Pant *primers amplified Common Leopard only; *Unci *primers amplified Snow Leopard only, and *Prio *primers amplified Cat Leopard only (Figure 2). Crossamplification through ecoPCR yielded identical results when allowing two mismatches. A more extensive analysis using ecoPCR, and allowing a larger number of mismatches (i.e., simulating more relaxed PCR conditions), shows that *Pant *primers require at least 3 mismatches for cross-amplifying *Uncia uncia*. Similarly, *Unci *and *Prio *primers require at least 4 mismatches for cross amplifying other species.

### Evaluation of vertebrate primer pairs: Taxonomic coverage

**Figure 3:**
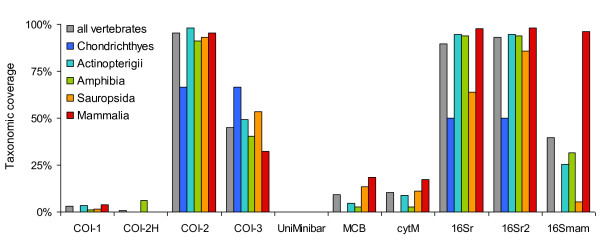
**Taxonomic coverage of different primer pairs tested over the reference database**.

**Figure 4:**
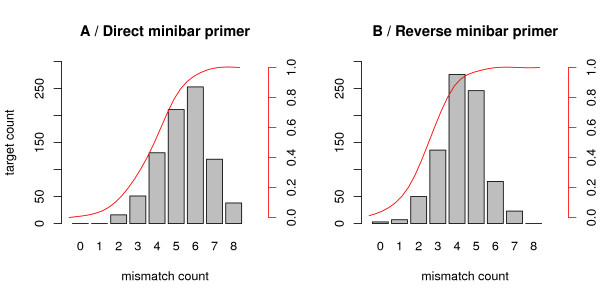
**Mismatches between Uni-Minibar primers and vertebrate sequences**. The histograms show the distribution of mismatch counts between (A) direct Uni-Minibar primer or (B) reverse Uni-Minibar primer and their target loci on mitochondrial DNA, as revealed by a ecoPCR run using Uni-Minibar primers to amplify our mitochondrial reference database. For this run, 8 mismatches were tolerated for each primer. The red curve and the associated right axis represent the cumulative fraction of amplified sequences with less that *m *mismatch. We can observe than a few species present a small count of mismatches; these sequences with a few mismatches are expected to be advantageously amplified in a DNA mix containing multiple species.

The primer pairs tested showed very different taxonomic coverage. Overall, COI-2, 16Sr and 16Sr2 were the primers with the highest percentages of species amplified (95, 90 and 93% of vertebrates amplified, respectively; Figure 3, table 2). Following our *in silico *PCRs, the primers with the lowest coverage corresponded to Uni-Minibar, COI-1, COI-2H, MCB and cytM. The primers also differed in their performance in amplifying the major clades of vertebrates. For example, COI-3 had the highest amplification rate in Chondrichthyes, while it amplified only 32% of the mammals. Conversely, 16Smam amplified most of the mammals, but failed in the amplification of Chondrichthyes (Figure 3, table 2). Nevertheless, in a similar way to how modifying the annealing temperature influences *in vitro *PCR [17], the number of electronically amplified species can be quickly increased by allowing a larger number of mismatches (Figure 4). For example, with primers Uni-Minibar, the proportion of amplified species reached 98% with eight tolerated mismatches (Figure 4).

**Table 2:** Taxonomic coverage and resolution capacity (B_S_) of the different barcodes tested.

	all vertebrates		Chondrichthyes		Actinopterigii		Amphibia		Sauropsida		Mammalia	
	Taxonomic coverage											
COI-1	0.03		0.00		0.03		0.01		0.02		0.04	
COI-2H	0.01		0.00		0.00		0.06		0.00		0.00	
COI-2	0.95		0.67		0.98		0.91		0.93		0.96	
COI-3	0.45		0.67		0.49		0.41		0.53		0.32	
Uni-Minibar	0.00		0.00		0.00		0.00		0.00		0.00	
MCB	0.09		0.00		0.05		0.03		0.14		0.18	
cytM	0.10		0.00		0.09		0.03		0.11		0.17	
16Sr	0.90		0.50		0.94		0.94		0.64		0.98	
16Sr2	0.93		0.50		0.94		0.94		0.86		0.98	
16Smam	0.40		0.00		0.25		0.32		0.05		0.96	

	Resolution capacity											
	B_S_	*N*	B_S_	*N*	B_S_	*N*	B_S_	*N*	B_S_	*N*	B_S_	*N*
COI-1	1.00	49	*	-	1.00	16	*	-	1.00	11	*	-
COI-2	0.97	2113	*	-	0.96	538	1.00	76	0.97	311	0.98	235
COI-3	0.96	650	*	-	0.94	326	1.00	33	0.96	159	1.00	75
MCB	0.95	1426	*	-	0.88	203	*	-	0.95	841	0.97	364
cytM	0.90	935	*	-	0.80	177	*	-	0.99	272	0.94	476
16Sr	0.98	1730	*	-	0.97	624	1.00	118	0.99	243	0.99	560
16Sr2	0.98	1769	*	-	0.97	624	1.00	118	0.99	286	0.99	560
16Smam	0.83	3242	*	-	0.83	518	0.76	1297	0.90	351	0.90	1063

In the analysis of Resolutions, only primers amplifying more than 10 species per taxon are considered.

N: number of sequences amplified from Genbank.

* The resolution was not calculated as the primer pairs amplified 10 or less different species for this taxon.

### Resolution capacity of barcode regions

**Figure 5:**
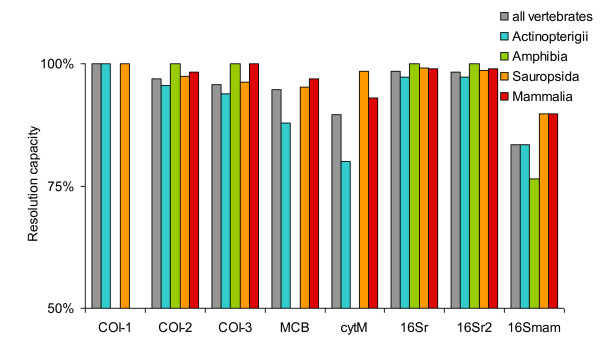
**Resolution capacity of barcodes tested over the entire GenBank**. Resolution is reported only for primer × taxon combinations that amplified more than 10 species. In all cases, resolution was > 50%.

When tested over the entire Genbank, most of the primer pairs had a very high resolution capacity, indicated by a high B_s _index (Figure 5; table 2). We did not calculate B_s _for primers Uni-Minibar and COI-2H because of the low number of species amplified with the settings used for this analysis (see discussion). Only the 16Smam primer pair, which amplifies a very short sequence (140 bp), had B_s _< 85%. B_s _was ≥ 90% for all other primer pairs and even > 97% for 16Sr and 16Sr2 whatever the vertebrate clade analysed (Figure 3, table 2). Apart from a few cases (e.g., low resolution of cytM within Actinopterigii), the resolution capacity of all primer pairs was consistently high across all taxa tested. These B_s _differences are not correlated with the number of Genbank sequences amplified (analysis over all vertebrates: Spearman's correlation *r*_S _= -0.323, *N *= 8, *p *= 0.4; the correlations between resolution and number of amplified sequences were not significant also within the monophyletic groups analysed).

The *in silico *PCRs performed over the entire GenBank always yielded sequences from the target mitochondrial region. None of the primers amplified sequences recorded as nuclear sequences in GenBank.

## Discussion

The identification of universal primer pairs amplifying fragments with high resolution capacity is a major task of DNA barcoding, and can help the broad scale analysis of life on earth. However, some authors argued that it is impossible that a single short sequence will be enough to distinguish all members of all species [12]. In this context, explicit *in silico *approaches like the one presented in this study allow analysing the properties of different sets of primers, and identifying the most appropriate ones *a priori*.

### In silico vs. real PCR

The real *in vitro *amplification pattern depends on PCR conditions. Controlling the PCR conditions can alter amplification results, and thus the taxonomic coverage of primers. For example, low annealing temperature and high concentration of MgCl_2 _reduce the specificity of primers in real-world PCR, and can thus allow amplification of target sequences with a larger number of mismatches in the primer regions [17]. Our *in silico *analyses have been performed allowing two mismatches. These parameters correspond well to actual amplification at rather high annealing temperatures (Figure 2), in accordance with previously published environmental genetics studies [22]. Nevertheless, these stringent conditions probably lead us to predict more false negative results (non electronic amplification of amplifiable sequences) than false positive ones (electronic amplification of non amplifiable sequences). Increasing the authorized mismatches can simulate more relaxed conditions, but the strict relationship between electronic and experimental conditions cannot be formally described. On the other hand, stringent PCR conditions reduce the risk of amplifying unwanted regions of the genome (see below), particularly when using degenerate primers. Furthermore, our study focused on *sensu lato *barcode primer pairs. These studies often amplify DNA extracted from environmental samples, which may represent a mix of the DNA of several taxa [6]. Considering this, primers and PCR conditions must be as specific as possible, because the rare species with a low number of mismatches in the primer region (Figure 4) are expected to be overamplified and overrepresented in the PCR products, while species that are present, but with a higher number of mismatches, may not be amplified enough to yield sequences. Therefore, "ideal" primers would have a constantly low number of mismatches, leading to a less biased estimate of species presence.

EcoPCR can also be used to simulate less stringent PCR conditions, allowing more mismatches. With this approach, primers can amplify a much larger number of species (Figure 3). For example, in our stringent *in silico *analysis the primers Uni-Minibar showed limited taxonomic coverage, and amplified very few vertebrates (table 2). Conversely, the PCRs performed by Meusnier *et al*. [20] showed that these primers can amplify nearly 100% of fish and Amphibians, at an annealing temperature of 46°C. Results coherent with Meusnier *et al*. [20] can be obtained using ecoPCR by allowing a large number of mismatches (up to eight) (Figure 4). Taking into account all these considerations, we have to assume that the taxonomic coverage B_c _estimated from ecoPCR is not an exact value, but it reflects the relative capacity of primer pairs to amplify a broad variety of taxa. For example, the fact that 16Sr amplifies a much larger number of species of amphibians than COI-2H [[11,23], see also [24] for a different approach] was correctly predicted by *in silico *analyses (see Figure 2, table 2).

Pseudogenes are a further potential issue in barcoding analysis; our approach may be affected by this trap. For instance, in our analyses none of the primers amplified nuclear sequences. However, nuclear sequences are underrepresented in Genbank; furthermore, the *in silico *amplification of pseudogenes would require the presence of a target nuclear sequence and both the corresponding primer regions, i.e., a good coverage of nuclear genome. Therefore it is difficult that ecoPCR hits nuclear pseudogenes, which can nevertheless be amplified by *in vitro *PCR, particularly under relaxed (e.g., low annealing temperature) conditions. Another potential issue of our approach is that the adjoining primer regions of sequences submitted to the databases are not a query-able portion of the database, therefore limiting the number of sequences obtained when ecoPCR is run over the entire GenBank. To partially address this issue, the assessment of taxonomic coverage was performed on species for which the whole mitochondrial genome was available, and therefore both target sequences and flanking regions are present. The increasing availability of whole mitochondrial genomes due the improvement sequencing technologies, and the rising of phylogenomics may reduce this limitation in the next future.

The correspondence between *in silico *and real PCR is certainly more accurate for the resolution capacity, still potential sources of bias remain. Our approach is based on the analysis of all the sequences deposited in GenBank, i.e., including thousands of vertebrate species in the example developed here. Assuming that all GenBank sequences are assigned to the correct species in the database, such approach uses the same kind of information than large scale barcoding studies. Clearly, the availability of sequences in different clades depends on the previous use of markers. For example, GenBank includes a very large number of COI sequences for Actinopterigii, while most of the mitochondrial sequences of mammals and amphibians are 16S. Furthermore, annotation errors are present in Genbank [25], and the error rate might be clade dependent. The B_S _index is sensible to these errors, leading to an underestimation of B_S_; therefore, as for B_C _previously, B_S _should be considered as a relative measure of primer performance.

### Comparison of vertebrate barcodes

Universality is a key feature of barcodes, and several strategies exist that can increase the taxonomic coverage of primer pairs. One strategy consists in making cocktails of degenerate primers. For example, the COI-2 primer pair [18] had one of the highest taxonomic coverages (figure 2). A predictable drawback of degenerate primers is a limited specificity with regards to the target DNA sequence amplified. However, our *in silico *PCRs performed on the whole GenBank did not amplify incorrect regions. All sequences amplified by the COI-2 primer pair were labelled in GenBank as mitochondrial COI, suggesting that these primers maintained enough specificity.

An alternative strategy consists in designing universal primers on highly conserved regions. This strategy has been used for example on the 16S, that exhibits some highly conserved regions in vertebrates [26]. The primers amplifying the 16S [[27,28]; this study] were very powerful, and had the highest taxonomic coverage and resolution capacity in vertebrates (Figure 2, Figure 3, table 2). The 16S region has been investigated as an alternate barcode locus for amphibians [11] but COI has not been rejected [24]. Some studies advocated that 16S has a too low rate of molecular evolution, and thus does not hold enough interspecific variation for a correct species identification [1]. Our analysis suggests that, at least in vertebrates, 16S has the same resolution capacity as COI, when using sequences with comparable length (500-600 bp), and therefore can be a good candidate site for barcoding. Nevertheless, the good performance of 16S observed in vertebrates may not be valid in other taxa; our *in silico *approach can be a key tool to analyse this possibility.

Long barcodes (500-600 bp) like the standard COI and 16S barcodes have a high resolution capacity, and are ideal candidates, for example, to unambiguously identify taxa in the context of the original DNA barcoding usage. However, studies analysing environmental samples or degraded DNA require the use of shorter DNA fragments [6,7,13,20,22,29] even though those smaller regions include less information. We have included in our analysis two primer pairs amplifying short sequences that can be used for such analyses: Uni-Minibar [20] and 16Smam [21], which amplify sequences of 130-140 bp. Our analysis did not amplify enough sequences to evaluate the overall performance of Uni-Minibar, but allowed estimating the taxonomic coverage of 16Smam, which was very high for mammals (i.e., the taxon for which the primers have been designed), and lower for the other clades (Figure 2). This short barcode had the lowest resolution capacity for identification at the species level (Figure 3). However, in many cases species identification is not needed in ecological barcoding, as information on the genus or family can be already valuable [6,7,13,29]. Indeed, the resolution of 16Smam was much higher if the aim was the identification at the genus or family level (resolution capacity of 96% and 100%, respectively; results not shown).

Our analysis focused on vertebrates, because several primers have been proposed for their *sensu lato *barcoding. Furthermore, the *in silico *assessment of primers strongly depends on the sequences in online databases; vertebrates are the phylum best covered by available sequences, therefore they are the ideal focus of a methodological analysis. Nevertheless, biodiversity on Earth is dominated by other phyla, such as arthropods and molluscs: The evaluation method describe here can be applied to these taxa and to any other ones, considering that the precision of the estimated B_S _and B_C _indices is directly linked to the amount and the quality of available sequences in public database corresponding to the studied clade.

## Conclusion

Based on our *in silico *analyses, the different barcodes tested showed dissimilar adequacy to be used according to the five clades of vertebrates studied. If we consider all possible applications of *sensu lato *barcoding, no single barcode could be identified as the best for all vertebrates. The primers amplifying COI-2 showed the highest taxonomic coverage in Actinopterigii and Sauropsida, while those amplifying 16Sr/16Sr2 showed the highest coverage of Amphibians and Mammals (Figure 3, table 2). Furthermore, the barcodes with the highest taxonomic coverage and resolution capacity (i.e., COI-2, 16Sr, 16Sr2) amplified long fragments, which can make their application problematic for describing biodiversity within environmental samples. In such a context, it is useful to select *a priori *the barcode that best suited the research topic. Our *in silico *method can help identifying the most appropriate barcode according to different aims. Such formal approach, which is possible thanks to the availability of bioinformatics tools and large public databases, can focus on target taxa or DNA regions and would make easier the validation of new barcodes by reducing the number of candidate primer pairs to be tested *in vitro*.

## Authors' contributions

GFF, EC, PT and FP participated to the design of the study; JB and EC developed ecoPcr; TR and EC developed Bs and Bc indices, WS performed *in vitro *experiments; GFF and SZ performed the analyses; GFF, EC, PT and FP wrote the paper. All authors read and approved the final manuscript.
